# Sustainable, Highly Efficient and Superhydrophobic Fluorinated Silica Functionalized Chitosan Aerogel for Gravity-Driven Oil/Water Separation

**DOI:** 10.3390/gels7020066

**Published:** 2021-06-02

**Authors:** Zhongjie Zhu, Lei Jiang, Jia Liu, Sirui He, Wei Shao

**Affiliations:** 1 Jiangsu Co-Innovation Center of Efficient Processing and Utilization of Forest Resources, Nanjing Forestry University, Nanjing 210037, China; 13382367651@163.com; 2 College of Chemical Engineering, Nanjing Forestry University, Nanjing 210037, China; 15655679696@163.com (L.J.); l17863961252@163.com (J.L.); he_1327313536@163.com (S.H.)

**Keywords:** superhydrophobicity, fluorinated silica, chitosan, oil adsorption, oil/water separation

## Abstract

A superhydrophobic fluorinated silica functionalized chitosan (F-CS) aerogel is constructed and fabricated by a simple and sustainable method in this study in order to achieve highly efficient gravity-driven oil/water separation performance. The fluorinated silica functionalization invests the pristine hydrophilic chitosan (CS) aerogel with promising superhydrophobicity with a water contact angle of 151.9°. This novel F-CS aerogel possesses three-dimensional structure with high porosity as well as good chemical stability and mechanical compression property. Moreover, it also shows striking self-cleaning performance and great oil adsorption capacity. Most importantly, the as-prepared aerogels exhibits fast and efficient separation of oil/water mixture by the gravity driven process with high separation efficiency. These great performances render the prepared F-CS aerogel a good candidate for oil/water separation in practical industrial application.

## 1. Introduction

In the wake of rapid developments in the economy and industry, industrial oil wastewater and organic solvents are massively discharged and oil spill accidents frequently occur. The existence of oils and organic pollutants in water leads to a series of environmental and ecological matters, thus threatening human health and aquatic organisms. Consequently, efficient implementation of the separation of oil/water mixtures is particularly important. The conventional techniques including oil booms/skimmers, microbiological deterioration, chemical coagulation or flocculation, chemical degradation, and ultrasonic separation have been used for oil/water separation applications [1,2,3,4]. These methods can partly realize the oil/water separation but they usually display low separation efficiency or high processing costs and even result in secondary pollution [5]. Therefore, materials with high oil absorption and separation ability are urgently desired in the oil/water mixture separation application. Since the oil/water mixture separation is based on the interface nature, the construction on surface special wettability could be effective and beneficial [3].

Water contact angle (WCA) with more than 150° on the surface is regarded to be a superhydrophobic surface, which has received a large number of studies in the research areas of self-cleaning, self-healing, anti-icing, anti-fouling, anti-corrosion, oil/water separation, and friction reduction [6,7,8]. Superhydrophobicity is usually a common accompaniment to suitable surface roughness with hierarchical microstructures/nanostructures. Superhydrophobic materials are very fit for oil/water separation applications because the oils can be filtered or be absorbed by the superhydrophobic material and those alongside the water are repelled, realizing the oil separation from oil/water mixture [9]. In addition, the wastewater containing various oils is usually under different corrosive environments, such as strong acidic/alkaline condition in practical industrial applications. Unfortunately, most of the current hydrophobic materials are unstable in these conditions. Therefore, superhydrophobic surfaces with low cost, easy operation, good recovery with high mechanical strength, excellent oil/water separation ability, and chemical stability are highly required [10]. Among the most studied hydrophobic materials, superhydrophobic aerogels show great advantages in the oil/water separation application.

Aerogels have been presumed to be one of the most cutting-edge candidates used in the area of oil/water mixture separation due to their three-dimensional (3D) networks. Their high porosity and large specific surface area favor oil absorption and storage. Moreover, aerogels exhibit ultralow density, recyclability, little secondary pollution, and etc. [11]. Biomaterial-based aerogels have been considered to be one of the most promising materials since they are rooted in natural plants and animal residues with renewable and environment-friendly properties. Chitosan (CS) is the deacetylated product of chitin that is derived from shrimp and crab shell. It can be used in the applications of drug delivery [12], wound dressing [13], food packaging [14], piezoresistive pressure sensor [15], and wastewater treatment [16,17]. CS/carboxymethyl cellulose/graphene oxide hybrid aerogel was fabricated and could be applied in pH-controlled drug delivery applications [12]. Novel alginate-CS aerogel fibers were obtained by scCO_2_ drying of a hydrogel and showed a strong antibacterial activity and promoted cell migration [13]. The CS/okra powder/nano-silicon aerogel composite films were prepared and displayed the potential to be utilized as edible food packaging films [14]. Hydrophobic modified CS aerogels have also been studied by many researchers. A conductive and superhydrophobic high-performance piezoresistive pressure sensor based on 1H,1H,2H,2H-perfluorooctyltriethoxysilane (FAS) modified reduced graphene oxide@carbon nanotubes/CS aerogel was successfully fabricated [15]. A stable GO/CS aerogel processed and exhibited high methylene blue adsorption capacity [17]. Superhydrophobic polydopamine/CS/reduced graphene oxide composite aerogel was prepared and exhibited high organic adsorption capacity [16].

Silica (SiO_2_) nanoparticles have been extensively applied in constructing hierarchical structures by using surface modification methods due to their high chemical stability, high specific surface area, and easy preparation. Moreover, the utilization of silica can provide a microscale/nanoscale rough structure on the surface with high WCA [18]. In order to further improve the hydrophobicity of the surface, decorating the silica on the surface of materials could be a satisfying choice. Many studies have been carried out to fabricate superhydrophobic surfaces. Polydimethylsiloxane/silica nanocomposite coatings were fabricated by a spray deposition method and showed superhydrophobic property with the hierarchical structures [19]. 1H,1H,2H,2H-perfluorodecyltrichlorosilane-functionalized silica based coating on printed circuit board showed good superhydrophobicity [20].

In this present work, micro/nano-binary hierarchical structures were designed and fabricated using triethoxy-1H,1H,2H,2H-tridecafluoro-n-octylsilane (FAS) modified silica nanoparticles as the superhydrophobic coatings on CS aerogel. The morphologies, chemical compositions, and mechanical compression properties were investigated. The contact angles and self-cleaning performances were evaluated. Finally, the oil absorption and oil/water mixture separation behaviors were determined.

## 2. Results and Discussion

### 2.1. SEM Morphologies

**Figure 1:**
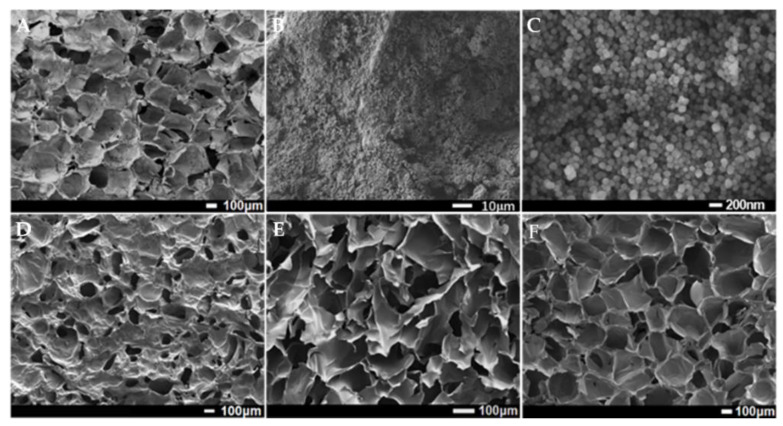
SEM images of surface morphology of F-CS aerogel (**A**–**C**) under different magnifications, surface morphology of CS aerogel (**D**), cross-sectional morphologies of CS (**E**), and F-CS aerogel (**F**).

The morphologies of the original CS aerogel and after surface functionalization were studied using SEM. The surface of CS aerogel has a porous morphology, as displayed in Figure 1D. After being functionalized with F-silica nanoparticles, a great number of nanoparticles covered the surface, which can be found in Figure 1A. It is noteworthy that the functionalized surface maintains its porous morphology with micro-sized pores. Meanwhile, Figure 1B,C are the high-resolution images of the F-silica nanoparticles on the surface of F-CS aerogel where no large aggregations of nanoparticles can be observed. The size distribution of F-silica nanoparticles is shown in Supplementary Figure S1 and it exhibits an average diameter of 110 nm with the particle size ranging from 80 nm to 140 nm. This rough micro- and nanostructure becomes the structural basis of hydrophobicity [21]. Figure 1E,F demonstrate the cross-sectional morphologies of CS aerogel and F-CS aerogel with the same three-dimensional structure with high porosity, demonstrating that the surface functionalization occurred only on the surface. It was reported that large pore size and high porosity of separation materials can provide great oil/water separation performance [22]. The element mappings with the combination of EDS spectrum (Figure S2) confirmed the existence of C, O, N, F, and Si elements on the surface of F-CS aerogel.

### 2.2. XPS and FTIR Analysis

**Figure 2:**
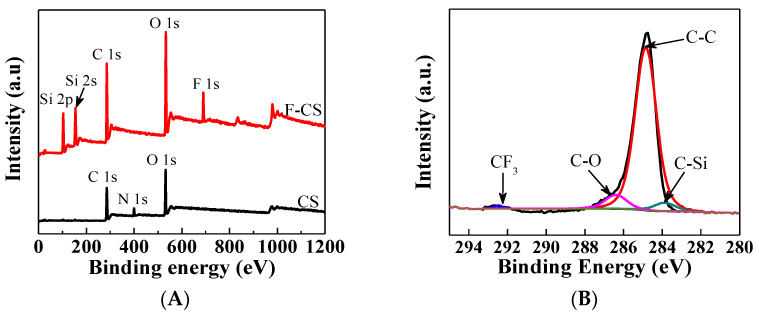
XPS survey spectra of CS and F-CS aerogels (**A**) and C 1s XPS spectrum of F-CS aerogels (**B**).

The existence of F and Si elements on the surface of F-CS aerogel was further confirmed by XPS. Figure 2A presents shows XPS survey spectra of CS and F-CS aerogels. In the spectrum of CS, XPS peaks that are at 532.5, 399 and 284.5 eV are related to O 1s, N 1s and C 1s, respectively, while a new peak is located at 690.3 eV corresponding to F 1s and two new peaks appearing at 152.4 and 104.3 eV are assigned to Si 2p [23]. The high-resolution C 1s spectrum of F-CS aerogel is shown in Figure 2B. There are four characteristic peaks at 293.8, 286.8, 284.8, and 284.0 eV attributing to the fluorocarbons in C-F_3_, C-O, C-C and C-Si groups, respectively [24,25,26].

**Figure 3:**
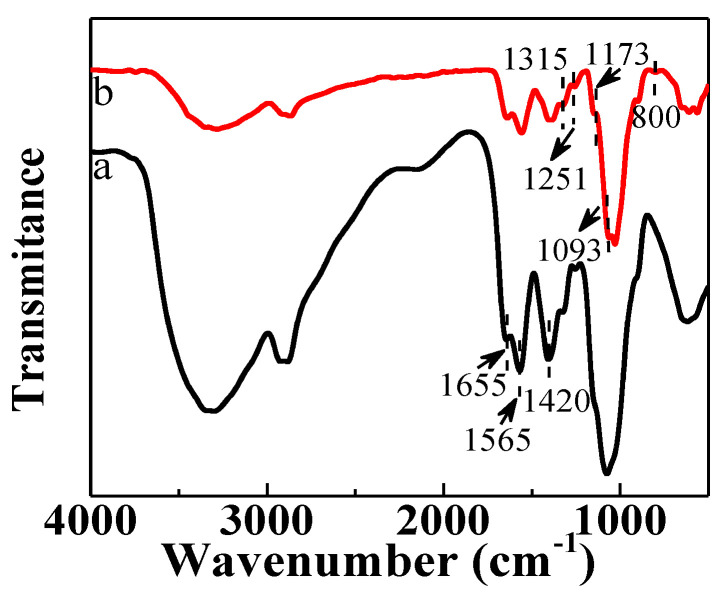
FTIR spectra of CS (**a**) and F-CS (**b**) aerogels.

The FTIR spectra of CS and F-CS aerogels are shown in Figure 3. In the spectrum of CS (curve a), a peak locating at 1655 cm^−1^ is attributed to the C=O in the amide group and the N=C of imine formed by crosslinking of CS with glutaraldehyde [27,28,29]. The absorptions at 1565 cm^−1^ and 1420 cm^−1^ are ascribed to the N-H bending vibration of the amine group and the N-H deformation vibration of amide [29]. In the spectrum of F-CS, absorption bands at 1093 cm^−1^ and 800 cm^−1^ arose, which were assigned to the asymmetric and symmetric vibrations of Si-O-Si of silica nanoparticles [30]. The peak at 1315cm^−1^ belongs to the CF_3_ and the absorption bands at 1251 cm^−1^ and 1173 cm^−1^ are assigned to the stretching vibrations of CF_2_ of FAS [31,32]. Based on the above results, the fluorinated silica nanoparticles were successfully grafted onto the surface of F-CS aerogel.

### 2.3. Mechanical Properties

**Figure 4:**
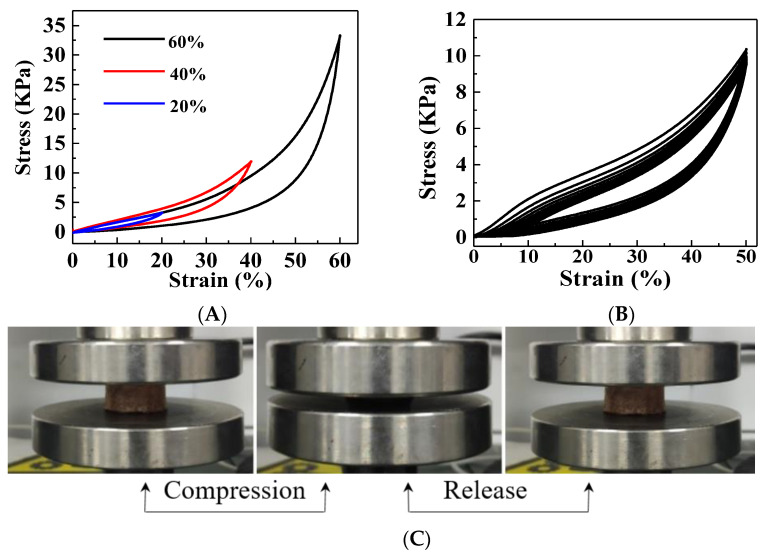
Compression stress-strain curves under 20%, 40%, and 60% strains (**A**), cyclic stress-strain curves at 50% strain for 10 cycles (**B**), and the pictures of the tenth cycle compress and release process at 50% strain of F-CS aerogel (**C**).

The compressibility of aerogels is usually considered to be a basic feature for the application in oil/water separation due to the fact that their compressibility performance determines their corresponding extrusion capacity [33]. The compressive loading–unloading curves of F-CS aerogel under strains of 20%, 40%, and 60% are displayed in Figure 4A. The same trend with three characteristic stages was exhibited in their stress-strain curves. (1) Elastic bending: The stress linearly dependent on strain that air escapes from the interspace of the porous aerogel in this linear elastic stage with the strain less than 10%. (2) Elastic buckling: The porous network collapses with the strain ranging from 10% to 45%. (3) Highly folded compaction: The porous network sequentially collapses to turn into a dense structure with the strain higher than 45%. The exhibited hysteresis lines reveal that there is energy dissipation during the loading and unloading process, which is mainly due to the buckling of the aerogel skeleton [34,35]. The 10 times cyclic compression of F-CS aerogel was studied under the strain of 50% and their stress-strain curves are illustrated in Figure 4B. The F-CS aerogel recovers well to return to its original shape after releasing the load with a slight plastic deformation after 10 cycles. In addition, the compression and recovering process of F-CS aerogel at the tenth cycle as depicted in Figure 4C displays that the cylindrical aerogel can recover to its initial shape rapidly after the loading release and this proves its good reversible compression capability. These results demonstrate that the prepared F-CS aerogel processes outstanding elasticity and mechanical stability and further shows the great potentials for their practical application in oil/water separation.

### 2.4. Surface Wettability

The surface wettability of the pristine CS and F-CS aerogels was investigated to study their hydrophobic and hydrophilic performance. Figure S3 displays the sequential WCA pictures of CS (A–C) and F-CS (D–F) aerogels. For the CS aerogel, a water droplet spreads and penetrates rapidly and completely into the CS aerogel in 0.5 s (pictures A–C in Figure S3), indicating the inherent hydrophilic nature of CS. On the other hand, the water droplet on the F-CS aerogel remains unchanged for 2 h, with the WAC of 151.9° (pictures D–F in Figure S3). Thus, the prepared F-CS aerogel displays superhydrophobic property with prominent water-repellent activity.

**Figure 5:**
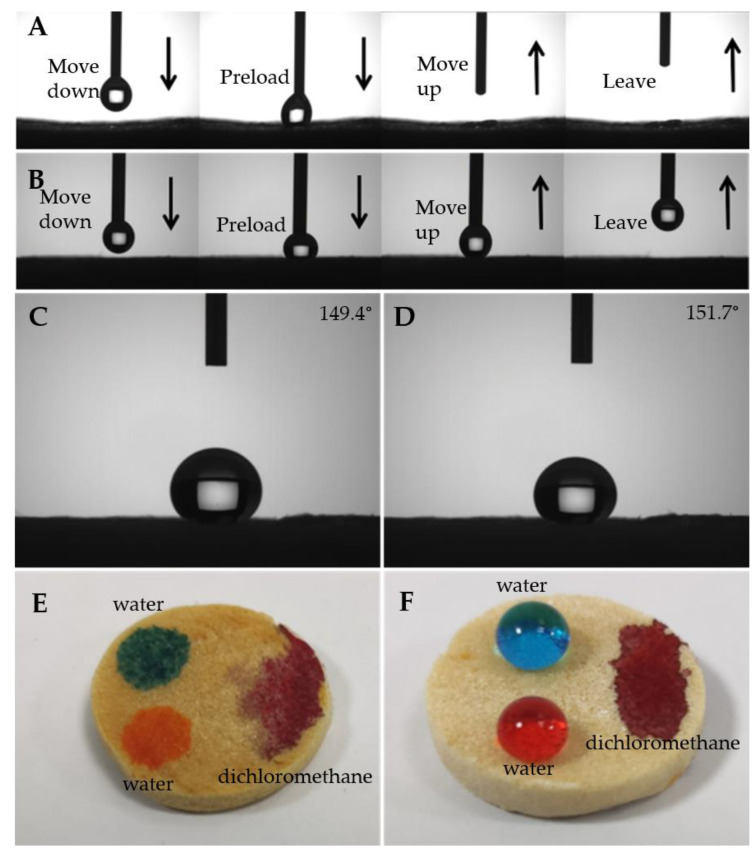
Dynamic water droplet adhesion and repelling test of CS (**A**) and F-CS (**B**), water contact angles of F-CS aerogel treated by different pH conditions (**C**: pH = 2 and **D**: pH = 12), and digital pictures of water (dyed with methyl blue and methyl orange) and dichloromethane (dyed with Oil red O) droplets on the surface of CS (**E**) and F-CS (**F**) aerogels.

Dynamic contact-detach water repellence test was carried out on CS and superhydrophobic F-CS aerogels. The water droplet adheres tightly to the surface of CS aerogel when adequately preloaded and then spreads rapidly when lifted (Figure 5A). On the other hand, the water droplet hardly adheres on the surface of F-CS aerogel after it is preloaded and the spherical water droplet, without any deformation, is preserved when it is lifted from the surface of F-CS aerogel (Figure 5B). This information illustrates that the prepared F-CS aerogel displays the excellent water repellence property and that it could repel water contact with the surface containing the oil and water mixture.

For the purposes of practical applications, the chemical stability of F-CS aerogel is important. It was conducted by immersing the F-CS aerogel into corrosive solutions (pH = 2 and 12) for 12 h. As illustrated in Figure 5C,D, the WCAs on the treated surface of F-CS aerogel (149.4° for acid treatment and 151.7° for alkaline treatment) maintain almost the same as that of pristine CS aerogel (151.9° as shown in the picture F in Figure S3). This information demonstrates that the F-CS aerogel possesses good chemical stability towards acidic and alkaline solutions with extraordinary superhydrophobicity. Figure 5E,F show blue and orange water droplets and red dichloromethane droplets on the surfaces of CS and F-CS aerogels, respectively. Both water and dichloromethane droplets spread and penetrate rapidly and completely on the surface of CS aerogel (Figure 5E). Therefore, the pristine CS aerogel does not exhibit any oil/water separation capability due to it possessing no wetting selectivity. Meanwhile, blue and orange waterdrops remain spherical on the surface of F-CS aerogel in comparison with the red dichloromethane droplet and spreads and penetrates into it. Consequently, the above results demonstrate that the F-CS aerogel exhibits superoleophilic and superhydrophobic characteristics.

### 2.5. Self-Cleaning Behavior of F-CS Aerogel

**Figure 6:**
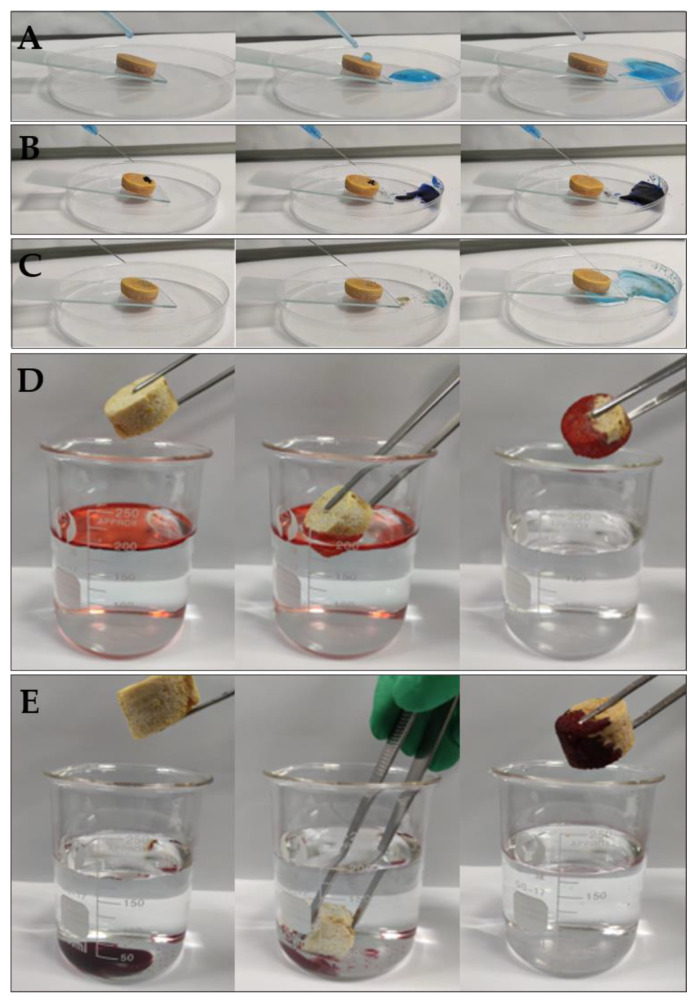
Methyl blue dyed water rolling off the surface (**A**), methylene blue powders (**B**) and sands washed away by running water (**C**), removal of petroleum ether (**D**), and dichloromethane (**E**) (dyed with Oil red O) from the surface and bottom of water with F-CS aerogel.

In order to further investigate the superhydrophobicity of the surface of F-CS aerogel, small droplets of methylene blue dyed water were dripped onto the surfaces. As illustrated in Figure 6A, the water droplet rolls off the surface rapidly without any trace left on it and confirms the superhydrophobicity of the prepared F-CS aerogel. Furthermore, the self-cleaning behavior was studied with methylene blue powders and sands as contaminants which were sprinkled onto the surface of F-CS aerogel. As it was shown in Figure 6B,C, the methylene blue powders and sands were washed away by running dyed water without any trace or stain on the surface of F-CS aerogel and leaves the original clean surface. The good self-cleaning property makes the aerogel protect the surface from pollutants in its practical application since the pollutants, such as dusts, could be washed away by water [36]. Therefore, the prepared F-CS aerogel possesses excellent superhydrophobicity and self-cleaning property.

### 2.6. Oil Adsorption Behavior of F-CS Aerogel

Since F-CS aerogel exhibits excellent intrinsic properties containing light weight and porous structure, great compressibility, and superhydrophobicity, it can be applied in the removal of oils from oil/water mixtures. Figure 6D,E show the application of F-CS aerogel for the removal of oil from water. As shown in Figure 6D, petroleum ether (dyed with Oil Red O) floating on water was rapidly and completely absorbed by F-CS aerogel which leaves clean water with no visible oil drops. In addition, dichloromethane was selected as the dense oil model. Figure 6E display the successful removal of dichloromethane (dyed with Oil red O) from water. Once F-CS aerogel contacted the dichloromethane sinking under the water, it absorbed dichloromethane quickly and perfectly as well. Thus, the F-CS aerogel can efficiently separate both light oils and dense oils from water. Furthermore, the collection of the absorbed oil is very simple through the crimping of the aerogel.

It is a meaningful parameter to evaluate the absorbents based on their absorption capacities. The oil absorption behavior of F-CS aerogel was determined using various oils and organic solvents with different densities including chloroform, dichloromethane, vacuum pump oil, vegetable oil, petroleum ether, and hexane. The absorption capacities are presented in Figure S5. The F-CS aerogel exhibits the best absorption capacity towards chloroform as it can absorb chloroform 18.02 times its own weight. Then, the adsorption ability of F-CS aerogel of other oils or organic solvents ranges from 10.49 to 17.34 times its own weight. It is also found that the absorption capacity of F-CS aerogel on different oils possesses a linear relation with the densities of oils and this relationship is shown in Figure S6. F-CS aerogel exhibits the apparent advantages of adsorbing higher density oils and its adsorption capability is the highest towards chloroform with the highest density, while it processes the lowest adsorption behavior on petroleum ether with the lowest density. In addition, it is worth noting that the absorption ability of F-CS aerogel is comparable to those of other reported silica functionalized oil and organic solvent absorbents, such as fiber/silica composite aerogel (nine times of its own weight) [37], SiO_2_/PU membrane (6.6 g/g dichloromethane and 2.4 g/g hexane) [38], and MCC/MC silica sponge (13 and 7 times its own weight towards chloroform and petroleum ether) [39].

### 2.7. Oil/Water Separation Performance of F-CS Aerogel

**Figure 7:**
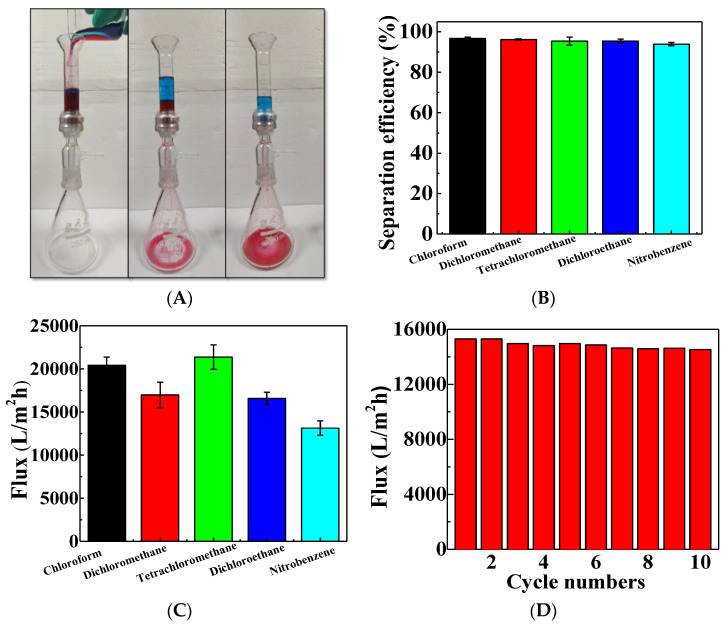
The processes of F-CS aerogel for separating a mixture of methylene blue dyed water and Oil red O dyed dichloromethane (**A**), separation efficiency (**B**), and separation fluxes of F-CS aerogel (**C**,**D**) driven by sole gravity.

A series of oil/water separation tests were carried out to assess the oil/water separation capability of the superhydrophobic F-CS aerogel. As it is displayed in Figure 7A, the aerogel was fixed between the glass tube and the conical flask with an inner diameter of 15 mm. Subsequently, oil red O dyed dichloromethane/methylene blue dyed water mixture was slowly added into the upper tube. It can clearly be seen that dichloromethane passes through the aerogel freely and drops into the ground flasks driven by gravity; this leaves the water retained above the aerogel because of the superhydrophobicity of the prepared aerogel. This result further indicates that F-CS aerogel exhibits great superhydrophobicity which could be beneficial for oil/water separation performance without any external driven force.

The oil/water separation performance of F-CS aerogel was further determined through the gauging of separation flux and computing its separation efficiency. The calculated separation efficiencies of various oil/water mixtures are shown in Figure 7B. The prepared F-CS aerogel presents relatively high separation efficiencies in the range of 94–97% for oil/water mixtures, indicating F-CS aerogel could separate various heavy oil/water mixtures effectively. Moreover, the separation fluxes for various heavy oils/water were calculated and listed in Figure 7C. The separation fluxes are 20,412, 16,972, 21,353, 16,572, and 13,136 L·m^−2^·h^−1^ for chloroform/water, dichloromethane/water, tetrachloromethane/water, dichloroethane/water, and nitrobenzene/water, respectively. The oil separation fluxes of reported silica functionalized materials are displayed in Table 1. As it is shown, the oil separation fluxes in our work are comparable with recently reported ones. In addition, the reuse possibility is confirmed by a stable oil/water separation activity after 10 cycles and the results are shown in Figure 7D. Thus, F-CS aerogel exhibits great oil/water separation performance with good reuse stability.

## 3. Conclusions

FAS modified silica nanoparticles have successfully been functionalized as CS aerogels with the average sizes of 110 nm. Superhydrophobic effects have been achieved with the WCA of 151.9° with good chemical stability. The fabricated F-CS aerogels show great mechanical compression properties with good recovery ability. In addition, the aerogels also exhibit great oil adsorption and self-cleaning performances. Most importantly, the F-CS aerogels possess highly efficient gravity-driven oil/water mixture separation capability with high separation flux and separation efficiency. Moreover, they also display good reuse stability. These results illustrate that the sustainable and superhydrophobic fluorinated silica functionalized CS aerogels exhibit great potential in the oil/water mixture separation application.

## 4. Experimental Section

### 4.1. Materials

Tetraethyl orthosilicate (TEOS) was purchased from Aladdin Bio-Chem Technology Co., Ltd. (Shanghai, China). FAS was purchased from Macklin Biochemical Co. Ltd. (Shanghai, China). CS, dichloromethane, chloroform, tetrachloromethane, nitrobenzene, dichloroethane, methylene blue, and oil red O were purchased from Sinopharm Chemical Reagent Co. Ltd. (Shanghai, China). Glutaraldehyde was purchased from Sigma-Aldrich. Acetic acid and ammonia were purchased from Nanjing Reagent Co. Ltd. (Nanjing, China). Petroleum ether was purchased from Wuxi City Yasheng Chemical Co. Ltd. (Wuxi, China). The n-hexane used was purchased from Shanghai Lingfeng Chemical Reagent Co. Ltd. (Shanghai, China). Vacuum pump oil (100#) was purchased from Lianya Chemical Co. Ltd. (Ningbo, China). Soya bean oil was purchased from Yihaijiali Food Co. Ltd. (Shanghai, China). All chemicals were used without any purification.

### 4.2. Synthesis of Fluorinated Silica Nanoparticles

Silica nanoparticles were synthesized by the sol–gel method according to the Stöber method [47]. Briefly, 9 mL of TEOS and 6 mL ethanol mixture were dipped into the solution including 8 mL of ammonia and 44 mL of ethanol solution with a rate of 0.25 mL/min. They were then stirred for 2 h and centrifuged at 10,000 rpm for 5 min to get precipitate. The precipitate was rinsed with ethanol and then centrifuged at 10,000 rpm for 5 min and this process was repeated three times. Finally, SiO_2_ was obtained after being dried in a vacuum at 65 °C for 10 h. The amount of 3 g SiO_2_ was added into 5 % wt ammonia solution and ultrasonic (200 W) for 30 min. The above suspension was added slowly into the FAS (0.6 mL)/ethanol (80 mL) mixture and was kept stirring at 40 °C for 24 h. They were then centrifuged at 10,000 rpm for 5 min to get precipitate. The precipitate was rinsed with ethanol and then centrifuged at 10,000 rpm for 5 min and this process was repeated three times. Finally, fluorinated silica nanoparticles (F-SiO_2_) were achieved after being dried in the vacuum drying oven at 65 °C for 10 h.

### 4.3. Preparation of the F-CS Aerogel

The amount of 10 mL 2% glutaraldehyde solution was added into pre-dissolved CS acetic acid solution (2 g CS in 90 mL 1% acetic acid). The mixture was kept at room temperature for 2 day to form hydrogel. The CS aerogel was obtained by freeze-drying at −40 °C for 24 h after the unreacted chemicals were removed by immersing them into de-ionized water for 2 d. Then, 1.4 g F-SiO_2_ was added into 20 mL tetrahydrofuran and ultrasonically treated for 30 min. Next, the CS aerogel was immersed into the above F-SiO_2_ suspension for 60 min and kept them in vacuum drying oven for 2 h. Then, it was rinsed with ethanol. The superhydrophobic aerogel was obtained after being dried in a vacuum drying oven for 10 h and noted as a F-CS aerogel.

### 4.4. Characterization

The morphologies of CS and F-CS aerogels were studied by JSM-7600F SEM. FTIR spectra were recorded using a Spectrum Two Spectrometer (PerkinElmer, Akron, OH, USA). XPS spectra were carried out with Thermo Escalab 250Xi instrument (Thermo Fisher, Waltham, MA, USA). Water contact angles were measured by a JC2000D contact angle analyzer (Powereach, Shanghai, China) with eight to ten contact angle measurements made on each sample. The compression test was performed on F-CS aerogel with disk shape (1 cm radius and 2 cm height) at a strain rate of 10 mm/min at room temperature by a dynamic mechanical analyzer (CMT4204, Shenzhen SANS, Shenzhen, China). Each sample was measured at least five times.

### 4.5. Oil/water Separation Performance

Different kinds of oils and organic solvents including chloroform, dichloromethane, vacuum pump oil, vegetable oil, petroleum ether, and n-hexane were applied in this study. The oil absorption capacity of F-CS aerogel was carried out by immersing them into various oils. The weight of the aerogel after achieving saturation was weighed immediately. The oil absorption capacity was calculated according to Equation (1):

H = m_1_/m_0_ × 100% (1)

where m_0_ and m_1_ are the weights of oils before and after adsorption, respectively.

The oil/water mixtures were prepared using 20 mL of oil red O dyed oils and 10 mL of methylene blue dyed water. The F-CS aerogel was placed between the Erlenmeyer flask and the vessels and further fixed with a metal clip. The oil/water mixtures were poured into the vessels and the gravity-driven oil/water separation ability is determined by calculating the oil permeation flux. The oil permeation flux (J, L·m^−2^·h^−1^) was calculated using the following Equation (2):

J = V/(A·T)(2)

where V represents the oil permeate volume (L) and A and T refer to the active area of the aerogel (m^2^) and the permeation time (h), respectively.

## Figures and Tables

**Table 1:** Performance comparison of the reported silica functionalized materials.

	Oil	Flux (L·m^−2^·h^−1^)	Ref
SNP/PBZ/PI	dichloromethane	~4800 (gravity)	Ma et al., 2019 [40]
PVDF/SiO_2_	chloroform	~2050 (gravity)	Gao et al., 2017 [41]
PC-a/SiO_2 0.6_	dichloromethane	~8470 (gravity)	Yao et al., 2019 [42]
PC-a/SiO_2 0.6_	chloroform	9320	Yao et al., 2019 [42]
FPMIA-1/SNP-2	dichloromethane	3311 (gravity)	Tang et al., 2013 [43]
PDA@SiO_2_ coated fabric	diesel oil	~4000 (gravity)	Guo et al., 2017 [44]
PS-CA-SiO_2_	petroleum ether	~2500 (gravity)	Xiong et al., 2020 [45]
PDMS–SiO_2_/PDA/paper	chloroform	~4600 (gravity)	Ruan et al., 2020 [46]
F-CS	dichloromethane	17,081 (gravity)	This work
F-CS	chloroform	20,401 (gravity)	This work

## Data Availability

The data presented in this study are available on request from the corresponding author.

## Supplementary Materials

The following are available online at https://www.mdpi.com/article/10.3390/gels7020066/s1. Figure S1: The size distribution of F-silica nanoparticles, Figure S2: Element mappings of C, O, N, F, Si and EDS spectrum of F-CS aerogel, Figure S3: The sequential WCA pictures of CS and F-CS aerogels, Figure S4: The collection process of the absorbed oil, Figure S5: The oil absorption weights of F-CS aerogel, Figure S6: The relationship between the oil density and the absorption weight of F-CS aerogel.
