# Supplementary material for: Sustainable, Highly Efficient and Superhydrophobic Fluorinated Silica Functionalized Chitosan Aerogel for Gravity-Driven Oil/Water Separation
Source: Gels. 2021 Jun 2;7(2):66. doi: 10.3390/gels7020066 (PMC8293147; doi:10.3390/gels7020066)
Supplement: Supplementary file 1 [file gels-07-00066-s001.zip › gels-1228587-supplementary.pdf]

Supplementary Materials

Sustainable, Highly Efficient and Superhydrophobic Fluorinated Silica Functionalized Chitosan Aerogel Gravity-Driven Oil/Water Separation

Zhongjie Zhu [1], Lei Jiang [2], Jia Liu [2], Sirui He [2] and Wei Shao [1,2,*]

[1] Jiangsu Co-Innovation Center of Efficient Processing and Utilization of Forest Resources, Nanjing Forestry University, Nanjing 210037, China; 13382367651@163.com
[2] College of Chemical Engineering, Nanjing Forestry University, Nanjing 210037, China; 15655679696@163.com (L.J.); l17863961252@163.com (J.L.); he_1327313536@163.com (S.H.)
* Correspondence: w.shao@njfu.edu.cn; Tel.: +86-25-85427024

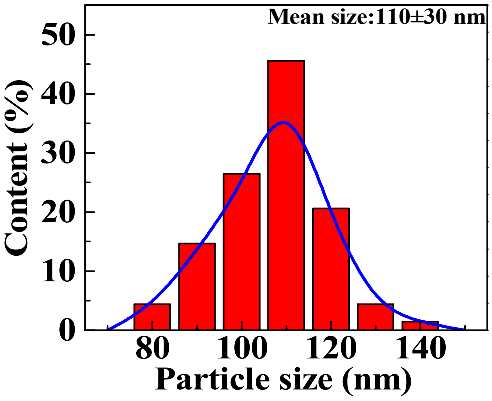

Figure S1. The size distribution of F-silica nanoparticles.

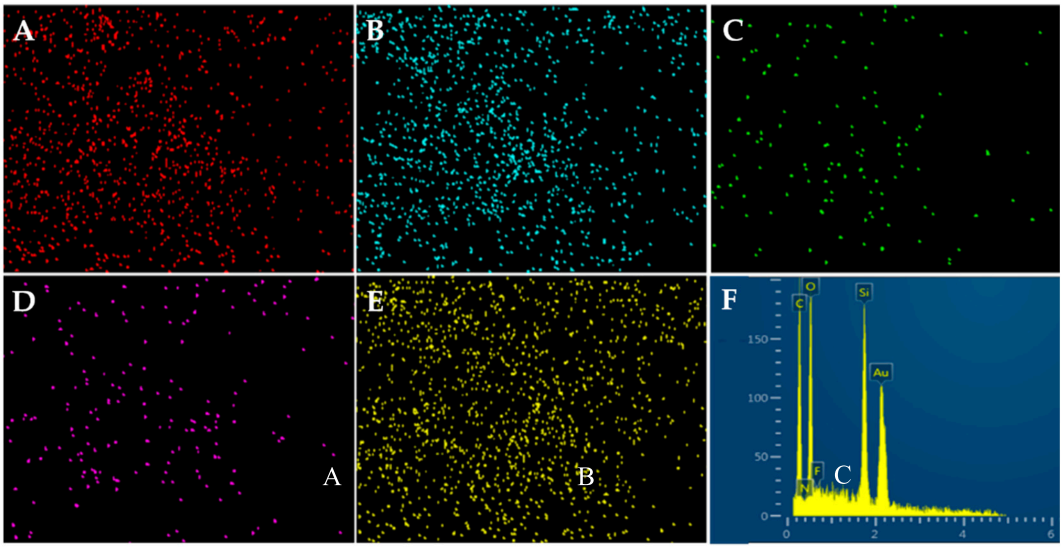

Figure S2. Element mappings of C (A), O (B), N (C), F (D), Si (E) and EDS spectrum (F) of F-CS aerogel.

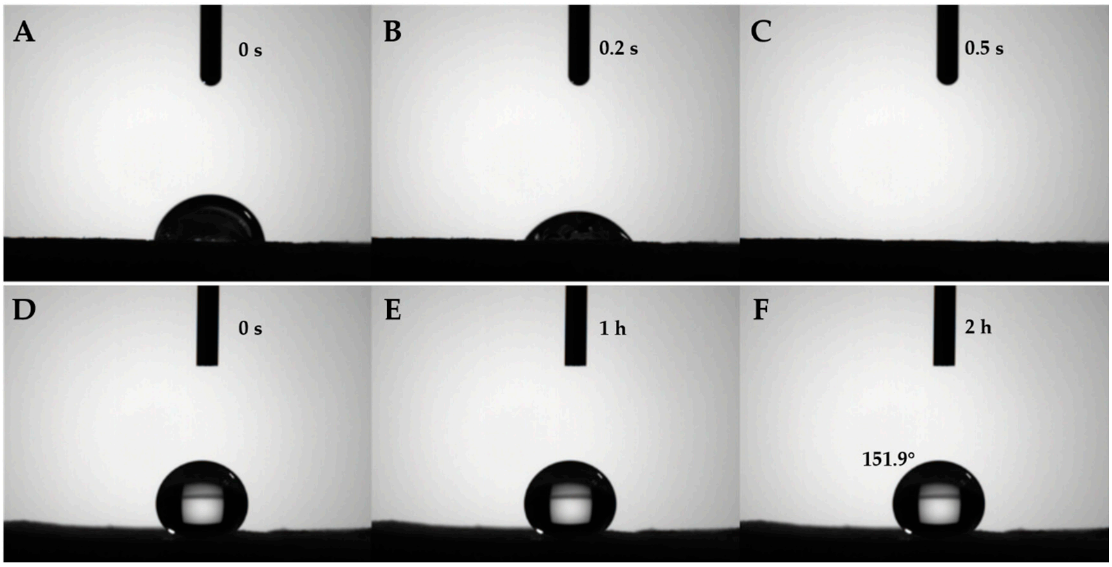

Figure S3. The sequential WCA pictures of CS (A-C) and F-CS (D-F) aerogels.

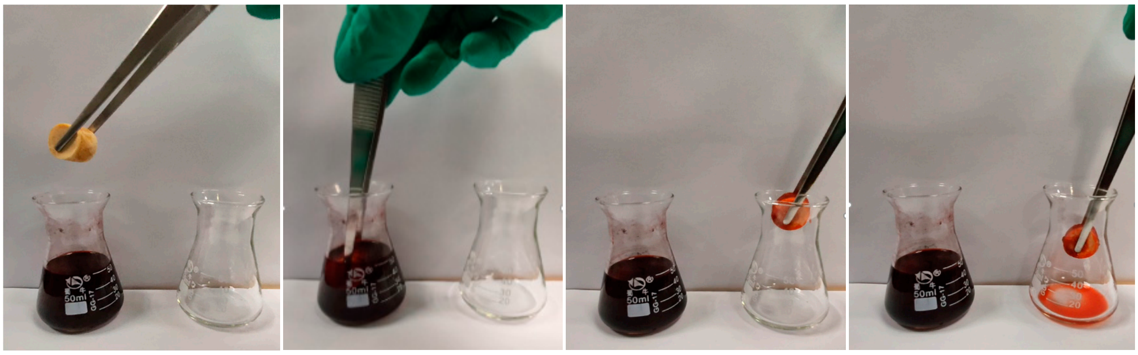

Figure S4. The collection process of the absorbed oil.

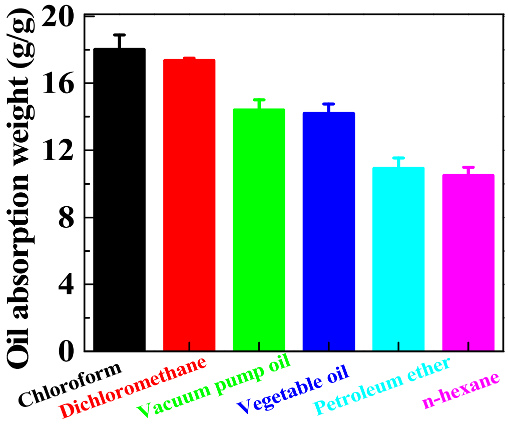

Figure S5. The oil absorption weights of F-CS aerogel.

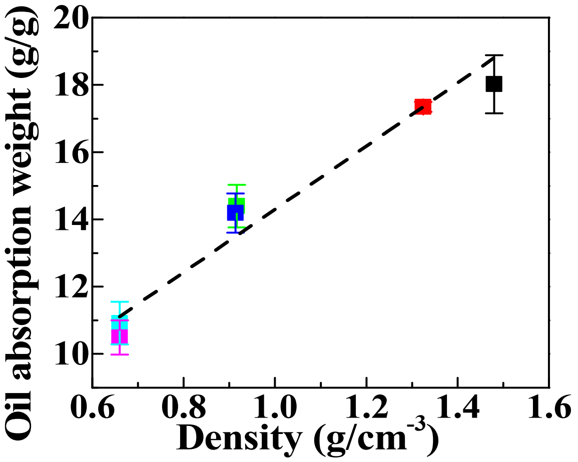

Figure S6. The relationship between the oil density and the absorption weight of F-CS aerogel.
